# Can viability assessment by DE-MRI predict survival in patients with multivessel disease and low ejection fraction? Influence of treatment strategies

**DOI:** 10.1186/1532-429X-13-S1-O90

**Published:** 2011-02-02

**Authors:** Bernhard L Gerber, Sylvie Ahn, Jean-Benoit le Polain de Waroux, Anne-Catherine Pouleur, Agnès Pasquet, Jean-Louis Vanoverschelde, Michel Rousseau

**Affiliations:** 1 Cliniques Universitaires St.Luc, Brussels, Belgium

## Introduction

Viability assessment by delayed-enhanced (DE) MR in patients with coronary artery disease (CAD) can predict improvement of ejection fraction (EF) after revascularization. However it is implication on survival is incompletely understood. Several studies suggested that large amounts of of DE, ie non-viable myocardium, increase risk of death. However these studies did not take into account the presence of CAD and and the potential protective effect of revascularization therapy on outcome. Also they conflict with earlier work employing nuclear imaging or Dobutamine Echocardiography who reported that large amounts of viable myocardium predict worse outcome if such myocardium is not revascularized.

## Purpose

The aim of the present work was to evaluate how viability by DE-MR predicts the survival in patient with low EF when revascularization of CAD is taken into account.

## Methods

We evaluated survival of patients who satisfied to Felkers definition of ischemic cardiomyopathy with EF<35% and who underwent DE-MR for assessment of myocardial viability and who had not been revascularized at the time of MR. The number of dysfunctional segments and the transmurality of DE was scored visually in 17 segments per patient. Kaplan-Meyer and Cox survival analysis was performed evaluating the predictive value of EF, volumes, treatment, and number of dysfunctional segments with necrosis

## Results

131 patients with a mean EF of 23±7% were followed. 7 pts. had one vessel disease (all proximal LAD disases), 35 2-vessel disease and 89 3-vessel disease. 75 patients underwent revascularization by CABG, and 23 by PTCA, while 34 remained under medical treatment. Follow-up was 100% complete at a median of 3.5 years. 43 pts died (all but 7 from cardiac cause) and 4 underwent heart transplantation. Four-year survival was significantly better in revascularized patients (75%) than in medically treated patients (46%, p<0.02 by log-rank test). Cox survival analysis demonstrated that only revascularization therapy was an independent predictor of survival. Presence of viability by DE-MR was not independently associated with survival either in revascularized or medically treated patients. Revascularization therapy significantly improved survival even in the absence of myocardial viability.

## Conclusions

In patients with ischemic cardiomyopathy, revascularization therapy is most important parameter associated with survival. Presence or absence of myocardial viability did not provide additional information on survival of patients. We thus conclude that all patients with ischemic cardiomyopathy will benefit from revascularization therapy, irrespectively of presence or absence of myocardial viability by DE-MR.

